# Effects of osteogenic growth peptide C-terminal pentapeptide and its analogue on bone remodeling in an osteoporosis rat model

**DOI:** 10.1515/med-2023-0656

**Published:** 2023-03-02

**Authors:** Yuhang Ma, Ying Zhang, Yi Lin, Xiaoying Ding, Yuntao Zhang

**Affiliations:** Department of Endocrinology and Metabolism, Shanghai General Hospital, Shanghai Jiao Tong University School of Medicine, Shanghai, 200080, China; School of Digital Construction, Shanghai Urban Construction Vocational College, Shanghai, 201999, China

**Keywords:** osteogenic growth peptide C-terminal pentapeptide, osteoporosis, bone mineral density, histomorphometry, biomechanical property

## Abstract

This study aimed to explore the effects of osteogenic growth peptide C-terminal pentapeptide (G36G), and its analog G48A on bone modeling in rats with ovariectomy-induced osteoporosis. Ovariectomized rats were administered PBS (OVX group), risedronate (RISE group), G36G combined with risedronate (36GRI group), G36G (G36G group), or G48A (G48A group). The sham-operation rats (SHAM group) were administered PBS. Serum osteocalcin and IGF-2 levels in the SHAM, OVX, G36G, G48A, and RISE groups were observably lower than the 36GRI group (*P* < 0.01) and the bone mineral density of the entire femur, distal metaphysis, and lumbar L1–L4 in the 36GRI group were notably increased (*P* < 0.05). The bending energy of the 36GRI group was prominently higher than the other groups (*P* < 0.05). Other features measured in the study that provided significant outcomes was the ratio of femora ash weight/dry weigh, parameters of trabecular bone volume (TBV)/total tissue volume, TBV/sponge bone volume, mean trabecular plate thickness, mean trabecular plate space, bone surface, parameters of sfract(s) and sfract(d), tetracycline-labeled, and osteoid surfaces. Bone loss in ovariectomized rats may be partially inhibited by G36G and G48A. A combination treatment with G36G and risedronate may be an effective intervention for osteoporosis.

## 1 Introduction

Osteoporosis is a common metabolic disorder characterized by impaired bone microarchitecture and reduced bone mass, leading to increased bone fragility and a high fracture risk [1,2]. Osteoporosis occurs when bone resorption exceeds bone formation. The incidence of osteoporosis increases with population aging, affecting approximately 30% of postmenopausal women [3,4]. With the acceleration of population aging, osteoporosis represents a major global health concern that has attracted increasing attention in recent years and imposes a heavy burden on patients and society. Osteoporosis is a multi-etiological disease, for which there is no effective treatment [5,6]. Therefore, the exploring of novel and efficient anti-osteoporosis drugs is an important research objective.

Osteogenic growth peptide (OGP) has attracted extensive clinical attention as a hematopoietic stimulator and a bone anabolic agent. OGP expedites osteogenic cell proliferation [7] and promotes fracture healing [8]. OGP (10–14) (G36G), the C-terminal pentapeptide cleaved from OGP (H-Tyr-Gly-Phe-Gly-Gly-OH), is the bioactive form of OGP that directly regulates the differentiation of progenitor rat bone marrow mesenchymal stem cells (BMSCs) into osteoblasts and adipocytes, thus playing a role in the treatment of osteoporosis [9]. A previous study also reported that G36G can be used managing different bone remodeling alterations, including glucocorticoid-induced osteoporosis [10]. G48A is an analog of G36G, and its resistance to enzymatic degradation can be enhanced by structural modification of an intramolecular site of G36G. G36G and its analog G48A have been reported to delay bone loss, promote bone mineral density (BMD), and increase bone biomechanical properties in ovariectomized rats [11]. However, the effects of G36G and its analog G48A on ovariectomy-induced osteoporosis in rats are largely unknown.

Herein we explored the effects of G36G and its analog G48A on biochemical and bone turnover markers, BMD, histomorphometry, and biomechanical properties in ovariectomized rats. Our efforts provide an experimental basis for new treatment strategies for osteoporosis.

## 2 Materials and methods

### 2.1 Animal grouping and interventions

All experimental procedures were performed in accordance with the guidelines of the Shanghai Science and Technology Council for Animal Care.

Sixteen weeks old female Sprague-Dawley rats (SLAC Laboratory Animal, Shanghai, China) were acclimated to the environment for 1 week and then randomly assigned into six groups (*n* = 6 per group): sham operation (SHAM), ovariectomized (OVX), risedronate (RISE), G36G + risedronate (36GRI), G36G, and G48A groups.

Rats were intraperitoneally injected with pentobarbital sodium (40 mg/kg body weight (BW); Sigma, Oakville, Ontario, Canada) for anesthetization and then subjected to bilateral ovariectomy (OVX). The SHAM group received the same procedure except for the OVX [12]. The ovariectomized rats were used as models of osteoporosis. Fifteen weeks post ovariectomy, the ovariectomized rats in the OVX, RISE, 36GRI, G36G, and G48A groups were subcutaneously injected with PBS (0.1 mL), risedronate (5 μg/kg BW), G36G (10 nmol/kg BW) combined with risedronate (5 μg/kg BW), G36G (10 nmol/kg BW), and G48A (10 nmol/kg BW), respectively, every day for 12 weeks. G36G and G48A were provided by Prof. Dexin Wang from the Institute of Materia Medica, Chinese Academy of Medical Sciences. Rats in the SHAM group underwent bilateral laparotomy and were subcutaneously injected with PBS (0.1 mL) daily for 12 weeks. All rats were double-labeled with tetracycline before euthanasia. The bilateral tibias were isolated and fixed with 70% ethanol. The lumbar vertebrae and femurs were separated, wrapped in saline gauze, and frozen at −20°C.

### 2.2 Baseline characteristics of rats

The BW and uterine weight of the rats were observed before and after the interventions. Biochemical indicators, including serum calcium (Ca), phosphorus (P), and alkaline phosphatase (ALP) levels, were measured before and after the interventions. Biochemical markers of bone turnover, including serum osteocalcin (BGP) and IGF-2 levels, were determined using BGP and IGF-2 detection kits (Science and Technology Development Center of the PLA General Hospital) after the interventions. The intra-assay variation in serum BGP was 2.61%, and the sensitivity was 0.22 μg/L. The intra-assay variation in serum IGF-2 was <10%, and the sensitivity was <0.1 ng/mL.

The dry and ash weights of the femurs were collected. To determine the dry weight, the femur was placed in a crucible and dried at 80°C for 2 h. The dried femur was then placed in a crucible and ashed at 600°C for 6 h.

The BMD of the entire segment of the isolated femur, distal metaphysis, and lumbar L1–4 was evaluated using dual-energy X-ray absorptiometry (DEXA) (LUNAR DPXIQ, GE Healthcare, USA). The intra-assay variation was 0.72% and the inter-assay variation was 0.84%.

### 2.3 Bone biomechanical parameters

Femoral biomechanical parameters were detected using a three-point bending test. The load measurement accuracy was 0.01 N, and the deflection accuracy was 0.001 mm. A small load of 200 N was used in this study. The elastic loading, bending energy, modulus of bending elasticity, stiffness coefficient, and maximal bending stress were measured using Bluehill software (Instron, Norwood, MA). The femora were kept moist at all times during the testing.

The anti-compression ability of the lumbar vertebrae was measured using a lumbar vertebral compression test. The vertebral body was prepared as a cylinder with two parallel planes at a height of approximately 5 mm. The loading speed was 2 mm/min, and the load-deformation curve was recorded. The strength and proportional limit, elastic modulus, maximum bone strain, and energy absorption were evaluated.

### 2.4 Morphological and dynamic parameters of tibial metaphysis

Bone slices were placed under an optical or fluorescence microscope, and the morphological and dynamic parameters of the tibial metaphysis were measured within the range of 1–3 mm distal to the epiphyseal plate line using an Opton Contron semi-automatic image processing system (Opton, Germany). The measured morphological parameters included total tissue volume (TTV), sponge bone volume (SBV), trabecular bone volume (TBV), TBV/TTV, trabecular area to volume ratio (S/V), TBV/SBV, mean trabecular plate thickness (MTPT), density (MTPD), and space (MTPS) were measured. The measured dynamic parameters included trabecular osteoid surface (TOS), percentage of tetracycline single- and double-labeled surface to trabecular bone surface (Sfract(s) and Sfract(d)), mean distance between tetracycline double-labeled lines (DDL), mineral appositional rate, and bone formation rate at tissue level (Svf).

### 2.5 Statistical analysis

For data conforming to a normal distribution, comparisons among groups were performed using analysis of variance (ANOVA). If the variance was homogeneous, comparison among groups was performed using the least significant difference (LSD) test; otherwise, Dunnett’s T3 test was applied. For non-normally distributed data, data were logarithmic transformed and conformed to normal distribution after transformation. If there was significant difference among groups (*P* value of ANOVA <0.05), the difference between the two groups was analyzed by post-hoc testing. Moreover, Bonferroni correction for multiple testing was applied, and an adjusted significance level was set as 0.0029 (0.05/15). The correlation between multiple variables was analyzed using partial correlation. Statistical analysis was completed using the SPSS17.0 software package (IBM, USA).

## 3 Results

### 3.1 Effects of different interventions on the baseline characteristics of rats

Fifteen weeks post ovariectomy, the BW of rats in the SHAM group was observably lower than that of ovariectomized rats in the OVX group (*P* < 0.01). After intervention for 12 weeks, the BW of rats in the SHAM group was also dramatically lower than that of ovariectomized rats in the OVX group (*P* < 0.01). However, the BW and ratio of uterine weight to BW after intervention did not differ significantly between the other intervention groups and OVX group (Table 1). The serum Ca, P, and ALP levels did not have difference between the two intervention groups (Table 2). Relative to the OVX group, the BGP level was significantly decreased in the G36G group (*P* < 0.05). Serum BGP and IGF-2 levels were obviously decreased in the SHAM, OVX, G36G, G48A, and RISE groups in comparison with those in the 36GRI group (*P* < 0.01) (Table 2). Taking BW as a covariate, intergroup covariance analysis revealed that the femur ash weight/dry weight ratio was not significantly different among the different intervention groups. The BMD of the whole femur, distal metaphysis, and lumbar L1–4 in the OVX group were all significantly lower than those in the SHAM group (*P* < 0.01). Relative to the OVX group, the BMD of the whole femur, distal metaphysis, and lumbar L1–4 in the 36GRI group and the BMD of the whole femur and distal metaphysis in the RISE group were observably increased (*P* < 0.05) (Table 3).

**Table 1:** The BW and uterine weight of rats in different intervention groups before and after interventions ( \bar{x}\pm s] )

Groups	BW before intervention (g)	BW after intervention (g)	Uterine weight (g)	Uterine weight/BW after intervention
G36G	376.0 ± 27.0^▲^	407.0 ± 29.9^▲^	0.115 ± 0.03^▲^	0.00029 ± 0.0001^▲^
G48A	382.5 ± 9.6^▲^	410.0 ± 14.1^▲^	0.109 ± 0.06^▲^	0.00027 ± 0.0001^▲^
RISE	367.5 ± 22.5^▲^	397.5 ± 31.0^▲^	0.260 ± 0.07^▲^	0.00044 ± 0.0002^▲^
36GRI	387.5 ± 21.0^▲^	417.5 ± 9.6^▲^	0.100 ± 0.07^▲^	0.00026 ± 0.0000^▲^
OVX	380.0 ± 36.7^▲^	401.2 ± 37.9^▲^	0.106 ± 0.05^▲^	0.00026 ± 0.0001^▲^
SHAM	300.0 ± 18.3	316.3 ± 22.3	0.607 ± 0.15	0.00192 ± 0.0004
*F* value	13.556	14.687	15.716	49.928
*P* value	0.000	0.000	0.000	0.000

Compared to OVX group, ^▽^ *P* < 0.05, ^▼^ *P* < 0.01; compared to SHAM group, ^△^ *P* < 0.05, ^▲^ *P* < 0.01.

**Table 2:** Serum Ca, P, ALP, BGP, and IGF-2 levels in different intervention groups after interventions ( \bar{x}\pm s] )

Groups	Ca (mmol/L)	P (mmol/L)	ALP (μ/L)	BGP (mmol/L)	IGF-2 (mmol/L)
G36G	2.91 ± 0.39	2.88 ± 0.33	65.5 ± 13.4	1.45 ± 0.19^■▽^	2.01 ± 0.10^□△^
G48A	2.59 ± 0.39	2.78 ± 0.73	64.0 ± 10.1	1.66 ± 0.04^□^	2.09 ± 0.58^□△^
RISE	2.75 ± 0.63	2.45 ± 0.17	71.5 ± 13.4	1.56 ± 0.22^■^	2.02 ± 0.49^□^
36GRI	3.02 ± 0.47	2.51 ± 0.26	78.7 ± 6.35	1.96 ± 0.16	2.38 ± 0.63
OVX	2.76 ± 0.40	2.61 ± 0.32	70.5 ± 13.1	1.72 ± 0.42^□^	2.14 ± 0.22^□^
SHAM	2.65 ± 0.22	2.25 ± 0.22	57.6 ± 18.4	1.58 ± 0.36^■^	1.83 ± 0.29^■▽^
*F* value	2.421	2.125	1.295	5.976	5.912
*P* value	0.103	0.138	0.334	0.005	0.006

ALP: alkaline phosphatase. BGP: osteocalcin. Compared to 36GRI group, ^□^ *P* < 0.05, ^■^ *P* < 0.01; compared to OVX group, ^▽^ *P* < 0.05, ^▼^ *P* < 0.01; compared to SHAM group, ^△^ *P* < 0.05, ^▲^ *P* < 0.01.

**Table 3:** Effects of different interventions on femur ash weight/dry weight ratio and BMD of whole femur, distal metaphysis, and lumbar L1–4 in rats ( \bar{x}\pm s] )

Groups	Femur ash weight/dry weight ratio	Distal metaphysis BMD (g/cm^2^)	Whole femur BMD (g/cm^2^)	Lumbar L1–L4 BMD (g/cm^2^)
G36G	0.612 ± 0.02	0.230 ± 0.01^▲■◣^	0.207 ± 0.01^▲◣■^	0.217 ± 0.01^△◢^
G48A	0.610 ± 0.01	0.234 ± 0.01^▲■◣^	0.215 ± 0.01^△◣□^	0.213 ± 0.00^▲◢^
RISE	0.617 ± 0.02	0.253 ± 0.01^▼^	0.229 ± 0.01^▼^	0.221 ± 0.01
36GRI	0.616 ± 0.01	0.253 ± 0.01^▼^	0.231 ± 0.01^▼^	0.231 ± 0.01^▽^
OVX	0.610 ± 0.02	0.226 ± 0.01	0.211 ± 0.01	0.214 ± 0.01
SHAM	0.631 ± 0.01	0.253 ± 0.01^▼^	0.225 ± 0.01^▼^	0.232 ± 0.01^▼^
*F* value	1.7	5.621	7.542	3.536
*P* value	0.175	0.002	0.000	0.016

BMD: bone mineral density. Compared to OVX group, ^▽^ *P* < 0.05, ^▼^ *P* < 0.01; compared to SHAM group, ^△^ *P* < 0.05, ^▲^ *P* < 0.01; compared to RISE group, ^□^ *P* < 0.05, ^■^ *P* < 0.01; compared to 36GRI group, ^◢^ *P* < 0.05, ^◣^ *P* < 0.01.

### 3.2 Effects of different interventions on bone biomechanical parameters

The three-point bending test revealed that the bending energy of the G36GRI group was prominently stronger than that of the other groups (*P* < 0.05). However, no significant differences were observed in the bending stress, modulus of bending elasticity, and stiffness coefficient among the different intervention groups (Table 4). The results of the lumbar vertebral compression test showed that the strength limit, proportional limit, maximum bone strain, elastic modulus, and energy absorption were not significantly different among the different intervention groups (Table 5).

**Table 4:** Effects of different interventions on the indexes of three-point bending test of femur in rats ( \bar{x}\pm s] )

Groups	Elastic loading (N)	Bending stress (MPa)	Bending energy (N/mm)	Modulus of bending elasticity (GPa)	Stiffness coefficient (N/mm^2^)
G36G	71.04 ± 8.19^■◣^	195.60 ± 52.1	16.19 ± 4.16^◣^	8.882 ± 2.84	40.32 ± 3.25
G48A	79.85 ± 2.36^◣^	161.27 ± 19.7	17.57 ± 2.91^◢^	7.163 ± 1.07	47.11 ± 9.02
RISE	86.37 ± 5.52	177.12 ± 22.4	18.83 ± 1.98^◢^	7.910 ± 0.96	50.50 ± 5.62
36GRI	87.73 ± 6.28^△^	196.28 ± 18.7	24.18 ± 5.92	7.911 ± 1.03	46.67 ± 6.07
OVX	78.34 ± 1.49	193.97 ± 31.4	19.03 ± 2.48^◢^	8.128 ± 1.73	41.54 ± 6.74
SHAM	77.52 ± 8.84	192.24 ± 27.3	17.51 ± 2.55^◣^	9.375 ± 1.92	43.54 ± 6.29
*F* value	3.862	0.845	2.813	1.030	1.624
*P* value	0.012	0.532	0.04	0.424	0.193

Compared to SHAM group, ^△^ *P* < 0.05, ^▲^ *P* < 0.01; compared to RISE group, ^□^ *P* < 0.05, ^■^ *P* < 0.01; compared to 36GRI group, ^◢^ *P* < 0.05, ^◣^ *P* < 0.01.

**Table 5:** Effects of different interventions on the indexes of lumbar vertebra compression test ( \bar{x}\pm s] )

Groups	Proportional limit (MPa)	Strength limit (MPa)	Energy absorption (N/mm)	Elastic modulus (GPa)	Maximum strain
G36G	12.43 ± 3.65	15.25 ± 4.02	16.34 ± 5.69	1.377 ± 0.97	0.020 ± 0.010
G48A	10.16 ± 2.06	13.89 ± 2.93	17.27 ± 7.34	0.954 ± 0.15	0.017 ± 0.005
RISE	12.87 ± 4.31	17.60 ± 6.01	19.45 ± 6.11	1.043 ± 0.50	0.022 ± 0.007
36GRI	12.98 ± 2.80	19.04 ± 2.80	21.94 ± 14.29	1.247 ± 0.35	0.022 ± 0.003
OVX	11.28 ± 5.06	16.24 ± 8.81	13.80 ± 3.89	1.184 ± 0.94	0.016 ± 0.004
SHAM	15.84 ± 2.96	20.23 ± 2.97	20.61 ± 9.04	1.622 ± 0.51	0.018 ± 0.004
*F* value	1.474	1.359	0.531	0.717	0.617
*P* value	0.242	0.276	0.750	0.618	0.645

Compared to OVX group, ^▽^ *P* < 0.05, ^▼^ *P* < 0.01; compared to SHAM group, ^△^ *P* < 0.05, ^▲^ *P* < 0.01.

### 3.3 Effects of different interventions on the morphological and dynamic parameters of tibial metaphysis

By analyzing the morphological parameters of the tibial metaphysis after different interventions, we found that the TBV/TTV and TBV/SBV were significantly lower and the MTPS was remarkably higher in the OVX group relative to those in the SHAM group (*P* < 0.01). In comparison to the OVX group, the TBV/TTV and TBV/SBV were significantly increased in the G36G, G48A, and 36GRI groups (*P* < 0.01), the S/V was dramatically decreased in the G36G and 36GRI groups (*P* < 0.05), the MTPT was remarkably promoted in the G36G and 36GRI groups (*P* < 0.05), and the MTPS was obviously decreased in the G36G, G48A, RISE, and 36GRI groups (*P* < 0.05) (Table 6). Further analysis of the dynamic parameters of the tibial metaphysis showed that Sfract(s) (%) was prominently decreased in the OVX group (2.38 ± 0.42) relative to that in the SHAM group (8.49 ± 2.94) and G48A group (10.87 ± 4.40) (*P* < 0.01), and no differences were observed in dynamic parameters among the other intervention groups (data not shown).

**Table 6:** Effects of different interventions on the morphological parameters of tibial epiphysis ( \bar{x}\pm s] )

Groups	TBV/TTV (%)	S/V (/mm)	TBV/SBV (%)	MTPT (μm)	MTPD (/mm^2^)	MTPS (μm)
G36G	10.15 ± 1.66^▲▼^	19.92 ± 3.66^◢▲▼^	15.42 ± 1.05^▲▼^	102.12 ± 18.75^◣▲▼^	1.55 ± 0.39^▲^	565.67 ± 148.49^◢▲▼^
G48A	8.49 ± 1.27^◢▽▲^	29.67 ± 2.45	12.26 ± 1.93^▲^	67.42 ± 2.35	1.82 ± 0.79^▲^	482.41 ± 87.790^▲▼^
RISE	7.42 ± 1.13^◣▲^	28.71 ± 1.73	10.42 ± 2.33^▲^	69.67 ± 3.31	1.50 ± 0.66^▲^	598.97 ± 105.42^◢▲▼^
36GRI	11.88 ± 2.63^▲▼^	25.43 ± 1.96^▽^	17.87 ± 2.89^◢▲▼^	78.64 ± 6.79^△▽^	2.27 ± 0.89^▲^	361.51 ± 72.540^△◢▼^
OVX	5.09 ± 1.32^▲^	31.85 ± 3.58	6.59 ± 2.56^▲^	62.80 ± 3.62	1.05 ± 0.23^▲^	889.79 ± 143.49^▲^
SHAM	21.19 ± 2.52	30.84 ± 4.21	29.89 ± 7.38	64.86 ± 2.59	4.61 ± 0.96	152.15 ± 60.85
*F* value	30.219	6.276	14.992	15.248	10.096	18.699
*P* value	0.000	0.004	0.000	0.000	0.001	0.000

TBV: trabecular bone volume; TTV: total tissue volume; S/V: total tissue volume; SBV: sponge bone volume; MTPT: mean trabecular plate thickness; MTPD: mean trabecular plate density; MTPS: mean trabecular plate space. Compared to OVX group, ^▽^ *P* < 0.05, ^▼^ *P* < 0.01; compared to SHAM group, ^△^ *P* < 0.05, ^▲^ *P* < 0.01; compared to 36GRI group, ^◢^ *P* < 0.05, ^◣^ *P* < 0.01.

## 4 Discussion

In the present study, we found that serum BGP and IGF-2 levels in SAHM, OVX, G36G, G48A, and RISE groups were significantly lower than those in 36GRI group. Compared with the OVX group, the BMD of the entire femur, distal metaphysis, and lumbar L1–4 in the 36GRI group was remarkably increased. Three-point bending test revealed that the bending energy of 36GRI group was prominently higher than other groups. Histomorphometry analysis revealed that multiple morphological parameters, including TBV/TTV, TBV/SBV, S/V, MTPS, and MTPT were altered after treatment of G36G, G48A, and 36GRI in ovariectomized rats, and only the dynamic parameter Sfract(s) of OVX group was significantly lower than that of SHAM and G48A groups.

Osteoporosis and its related fractures are associated with substantial morbidity and mortality and represent a public health concern worldwide [13,14]. Due to several associated limitations, such as poor bone targeting, off-target side effects, and low bioavailability, the treatment of osteoporosis with existing drug formulations remains challenging [15]. Currently, interventions for osteoporosis include two major strategies: inhibiting bone resorption and promoting bone formation [16]. Antiresorptive drugs such as bisphosphonates and estrogen can disrupt the biological behavior of osteoclasts to suppress bone resorption, whereas anabolic treatments such as parathyroid hormone analogs and growth factors can increase the bone remodeling rate to promote bone formation [17]. Therefore, the development of promising interventions to stimulate bone formation facilitates the treatment of osteoporosis and related fractures, consequently improving the quality of life of patients with osteoporosis.

OGP is a growth factor related to bone repair and regeneration [18], which has been reported to promote the proliferation and differentiation of osteoblasts and to increase whole-body bone quantity *in vivo* [19]. Moreover, both OGP and G36 promote new bone formation by modulating BMSCs differentiation into osteoblasts [9,20]. An *in vivo* study revealed that OGP acts as a hematopoietic stimulator, and OGP-loaded membranes could promote bone tissue formation and may be used as potential candidates for guided bone regeneration [21,22]. A recent study revealed that OGP released from an amphiphilic peptide (NapFFY) supramolecular hydrogel can promote osteogenesis and the reconstruction of bone tissue [23]. These data suggest that OGP is an attractive new anti-osteoporosis drug.

Animal models of osteoporosis are effective tools for investigating new prevention and treatment modalities. The *in vivo* effect of OPG in bone information was first described by Bab et al. [24], and they found that intravenous administration of OGP into adult male rats increased trabecular bone mass in the mandibular condyles. Chen et al. reported that OGP and G36 can reverses the majority of trabecular bone loss in ovariectomy-induced mice model [25]. Consistent with this study, we also used ovariectomized rat model to explore the effect of G36G and its analog G48A on bone modeling. The ovariectomized rat model is considered the first choice and most common model for such studies [26]. The ovariectomized rat model of osteoporosis mimics the bone loss caused by estrogen deficiency and exhibits clinical manifestations of postmenopausal osteoporosis [27]. The ovariectomized rat model has been employed to examine the effects of therapeutic agents such as bisphosphonates for managing osteoporosis [28]. Herein an ovariectomized rat model was also selected to explore the intervention effects of G36A and its analog G48A. To eliminate the influence of BW on bone metabolism indices as much as possible, a control group was set up in the experiment, and statistical methods of covariance analysis or partial correlation analysis with BW as a control variable were used in data processing. Our results showed that the rats in each group had better tolerance before and after intervention. Relative to the SHAM group, the uterine weight/BW ratio, femur ash weight/dry weight ratio, BMD, and histomorphometry of the ovariectomized rats suggest that the ovariectomized rat model of osteoporosis is reliable.

Biochemical bone turnover markers reflect changes in the bone metabolic microenvironment. Serum BGP levels are widely applied as markers of bone formation [29]. Singh et al. revealed that BGP had the potential as a predictor and surrogate marker of osteoporosis or fractures [30]. IGF-2, a major member of the IGF family, is frequently involved in bone metabolism [31]. Tsiridis et al. demonstrated that IGF-2 played a role in the differentiation of BMSCs into osteoblasts, as well as the proliferation of osteoblasts [32]. Furthermore, BMD has great significance in the early diagnosis of osteoporosis, prediction of fracture risk, and evaluation of intervention measures. Our results showed that serum BGP and IGF-2 levels and the BMD of the entire femur, distal metaphysis, and lumbar L1–4 in the 36GRI group were remarkably increased relative to the OVX or risedronate groups. These data hint that bone remodeling was degraded in the OVX group, whereas bone remodeling was partially recovered in the 36GRI group. The combination treatment with G36G and risedronate may be more beneficial for bone remodeling than the respective single intervention.

In addition to serum biochemistry and BMD, we evaluated mechanical strength and histomorphometry. To study the effects of treatment strategy during the repair process of bone defects, evaluation of structural fracture resistance is essential, and the ability of bone to resist fracture can only be evaluated by biomechanical strength tests. Three-point bending of the femur and lumbar vertebra compression tests are frequently used to assess bone mechanical strength and quantify biomechanical quality [33]. Three-point bending test showed that only the bending energy of the G36GRI group was significantly higher than that of the OVX group. We thus speculate that the combination of G36G and risedronate may improve the biomechanical properties of the bone to a certain degree. In addition, bone histomorphometry can intuitively observe subtle pathological changes in bone tissue, measure morphological parameters and kinetic parameters, and accurately judge the state of bone formation, bone absorption, and bone mineralization at an early stage. Our data showed that multiple morphological parameters for histomorphometry, including TBV/TTV, TBV/SBV, S/V, MTPS, and MTPT, were altered after treatment with G36G, G48A, and 36GRI in ovariectomized rats. These data suggest that G36G and its analog G48A may partially inhibit bone loss and promote bone modeling in an ovariectomized rat model of osteoporosis.

However, only female rat model of osteoporosis was used and the effects of G36G and its analog G48A on bone modeling in male animal model of osteoporosis were not investigated, which was a limitation of our study. In previous studies, the use of intravenously/subcutaneously administrated OGP has been extensively evaluated in male animal model. For instance, Brager et al. revealed that subcutaneous administration of OGP into male rats promoted an earlier bone-repair callus in femoral fracture [34]. Gabet et al. demonstrated that intravenous administration of OGP improved callus formation and function in femoral fracture in the male rat model [35]. Fei et al. indicated that OGP might increase the bone formation in OPG-deficient male mice via stimulating the proliferation of BMSCs [36]. These results suggest the clinical application of OGP for promoting bone formation in osteoporosis treatment and fractures repair. Further studies are still needed to investigate the effects of G36G and G48A on bone modeling in the male animal model of osteoporosis.

## 5 Conclusion

In conclusion, our findings reveal that bone loss in an osteoporosis rat model may be partially inhibited by G36G and its analog G48A. Combination of G36G and risedronate may be an effective intervention for osteoporosis. Despite these findings, it cannot be concluded with certainty that G36G and its analog G48A have a remarkable improvement in bone biomechanical properties during short-term therapy.
